# Association between intraoperative remifentanil use and postoperative hyperalgesia in adolescent idiopathic scoliosis surgery: a retrospective study

**DOI:** 10.1186/s12871-023-02127-8

**Published:** 2023-05-24

**Authors:** M. Shahnaz Hasan, Norashekeen Abdul Razak, Hing Wa Yip, Zheng-Yii Lee, Chris Yin Wei Chan, Mun Keong Kwan, Chee Kidd Chiu, Siti Nadzrah Yunus, Ching Choe Ng

**Affiliations:** 1 Department of Anesthesiology, Faculty of Medicine, University of Malaya, Kuala Lumpur, Malaysia; 2 Department of Orthopedic Surgery (NOCERAL), Faculty of Medicine, University of Malaya, Kuala Lumpur, Malaysia; 3 Department of Cardiac Anesthesiology and Intensive Care Medicine, Charité Berlin, Berlin, Germany

**Keywords:** Opioid, Remifentanil, Pain, Postoperative, Hyperalgesia, Scoliosis, Adolescent

## Abstract

**Background:**

The liberal use of remifentanil in spine surgery has been associated with an increased incidence of postoperative hyperalgesia. Nevertheless, controversies remain as the existing evidence is inconclusive to determine the relationship between remifentanil use and the development of opioid-induced hyperalgesia. We hypothesized that intraoperative infusion of higher dose remifentanil during scoliosis surgery is associated with postoperative hyperalgesia, manifesting clinically as greater postoperative morphine consumption and pain scores.

**Methods:**

Ninety-seven patients with adolescent idiopathic scoliosis (AIS) who underwent posterior spinal fusion surgery at a single tertiary institution from March 2019 until June 2020 were enrolled in this retrospective study. Anesthesia was maintained using a target-controlled infusion of remifentanil combined with volatile anesthetic desflurane in 92 patients, while five patients received it as part of total intravenous anesthesia. Intravenous ketamine, paracetamol, and fentanyl were administered as multimodal analgesia. All patients received patient-controlled analgesia (PCA) morphine postoperatively. Pain scores at rest and on movement, assessed using the numerical rating scale, and the cumulative PCA morphine consumption were collected at a six-hourly interval for up to 48 h. According to the median intraoperative remifentanil dose usage of 0.215 µg/kg/min, patients were divided into two groups: low dose and high dose group.

**Results:**

There were no significant differences in the pain score and cumulative PCA morphine consumption between the low and high dose remifentanil group. The mean duration of remifentanil infusion was 134.9 ± 22.0 and 123.4 ± 23.7 min, respectively.

**Conclusion:**

Intraoperative use of remifentanil as an adjuvant in AIS patients undergoing posterior spinal fusion surgery was not associated with postoperative hyperalgesia.

## Background

Remifentanil is a short-acting opioid with a predictable and rapid recovery profile, independent of the dose. These unique pharmacokinetic properties favour its liberal use as a manual or target-controlled infusion (TCI) in total intravenous anaesthesia (TIVA) or in combination with the volatile anaesthetic.

Remifentanil is widely used in spinal deformity surgeries, where rapid recovery, immediate postoperative neurological assessment, intraoperative hemodynamic stability, and minimal intraoperative interference with neurophysiological monitoring are prudent [1]. However, the use of remifentanil raised significant concerns among clinicians as its use had been repeatedly associated with the development of opioid-induced hyperalgesia (OIH), leading to increased postoperative pain and morphine usage.

OIH is defined as a state of nociceptive sensitization characterized by a contradictory response, whereby a patient receiving opioids for pain treatment might have an increased sensitivity to painful stimuli [2]. The incidence of OIH in the clinical settings can be assessed using several methods. This includes measuring the degree of pain intensity, amount of opioid consumption [3, 4], evaluation of secondary hyperalgesia using monofilaments and other sensory tests such as cold, heat, vibration, and electrical stimulation [5]. The development of OIH may cause several undesirable issues. It causes patient discomfort and delays mobilization and recovery after surgery; thus, prolonging the hospital stay. Moreover, it results in greater analgesics usage and associated side effects [6].

While the incidence of remifentanil-induced hyperalgesia in animals is well established [7], the literature surrounding its occurrence in humans is mixed, with evidence both supporting and refuting its association [8]. Most studies on humans found that hyperalgesia was induced during and after remifentanil infusion, especially with higher doses, longer duration of infusion, and abrupt changes in concentrations [9].

Posterior spinal fusion in idiopathic scoliosis is one of the most painful surgeries experienced by children and usually requires multimodal approach to pain management. The primary objective of this study is to explore the relationship between remifentanil and postoperative total morphine consumption and pain score. We hypothesize that a higher dose of remifentanil administered during scoliosis surgery is associated with the development of OIH, manifesting clinically as greater postoperative morphine consumption and pain scores.

## Methods

This retrospective single-centre observational study was conducted in a tertiary care centre and has been approved by the institutional ethics committee (MREC ID: 2021516-10137). All patients with adolescent idiopathic scoliosis (AIS) who underwent elective single-stage posterior spinal fusion surgery between March 2019 and June 2020 were reviewed for eligibility.

Inclusion criteria were age between 10 and 18 years and American Society of Anesthesiologists (ASA) physical status I to II. Exclusion criteria were patients on long term opioids or sedative drugs, known allergies to morphine or remifentanil, communication barrier, inability to self-administer morphine using patient-controlled analgesia (PCA) device, obesity (body mass index ≥ 30 kg/m^2^), renal and hepatic dysfunction, and incomplete medical record.

### Anaesthesia protocol

The provision of anaesthesia in our centre is by a dedicated team based on a standard perioperative protocol. General anaesthesia was induced with intravenous (IV) propofol 2–4 mg/kg, IV rocuronium 0.9 mg/kg, and TCI remifentanil 1–5 ng/ml to facilitate endotracheal intubation. Patients were ventilated with a 50% oxygen/air mixture.

Anaesthesia was maintained using a TCI of remifentanil between 2 and 8 ng/ml in combination with volatile anaesthetic desflurane in 92 patients at a Minimum Alveolar Concentration of 0.6 to 0.8 (approximate end-tidal desflurane of 4.8–5.2). In contrast, five patients received it as part of TIVA. Intraoperative monitoring included continuous invasive arterial blood pressure, heart rate, pulse oximetry, 3-lead electrocardiogram, and somatosensory evoked potential. Patients requiring motor evoked potential monitoring received TIVA. Intraoperative cell salvage technique was used as a blood conservation strategy and Hartmann’s solution as maintenance fluid therapy. Apart from TCI remifentanil, patients received the following intraoperative analgesics: IV ketamine 0.50 mg/kg and IV paracetamol 15.00 mg/kg before surgical incision, IV morphine 0.10–0.15 mg/kg 45 min and IV fentanyl 1.00 µg/kg 10 min before the end of surgery respectively.

To prevent postoperative nausea and vomiting, all patients received IV dexamethasone 0.10 mg/kg at induction and IV ondansetron 0.10 mg/kg at the end of surgery. At the end of the operation, TCI remifentanil was tapered off, and patients were extubated after meeting the criteria for extubation. In the post-anaesthesia care unit (PACU), patients were connected immediately to PCA morphine with the following protocol (bolus 1 mg, lock-out 5 min, without basal infusion, and 4 h limit is set at 20 mg).

Postoperatively in the ward, patients continued to receive PCA morphine (for the next 48 h), regular oral paracetamol 15 mg/kg six hourly, and oral Celecoxib 200 mg once or twice daily up to hospital discharge. After cessation of PCA morphine, all patients were prescribed subcutaneous morphine 5 mg as rescue analgesia.

### Surgical protocol

The Cobb angle was measured by drawing a line which was parallel to the upper end plate of the upper end vertebrae as well as the lower end vertebrae. Measurement of the Cobb angle was performed in the PACS system in our hospital computer system after magnification and windowing adjustment was performed at the intended images to improve the accuracy of the measurement. All the surgeries were performed by the two senior surgeons utilizing a dual attending surgeon strategy. All pedicle screws construct was used. Pedicle screws were inserted strategically at the base and proximal ends to provide a strong foundation for the construct. In between, alternate level screw placement was used, and additional screws might be inserted where deemed necessary to increase correction or to strengthen the construct. Radical facetectomies were performed to increase the spinal flexibility prior to the correction process. No Ponte osteotomies were performed. Fusion was augmented using local autogenous bone graft. Cell salvage autologous blood recovery system was used in all cases (Haemonetics Cell Saver 5 +). All patients had subfascial drains inserted prior to closure.

### Data acquisition

Patients’ demographics and operative data were retrieved, including intraoperative remifentanil usage in µg/kg/min as documented in the anaesthesia form.

Data on postoperative pain scores at rest and on movement (lateral turning) using numerical rating scale (NRS) of 0–10 were collected every six hours for a total of 48 h. The cumulative weight-adjusted morphine consumption through six hourly periods up to 48 h following surgery was calculated as the sum of the PCA morphine (mg) self-administered in the respective time frame after surgery divided by the body weight. All postoperative outcome data were collected by a recovery nurse and staff nurses on the ward. All of the data were retrieved, tabulated, and analysed.

### Statistical analysis

This is an exploratory observational study without a priori defined primary outcome. Based on feasibility consideration, a convenient sample size of 97 patients was enrolled; they were eligible cases between March 2019 to June 2020 in our centre [10]. The patients were divided into two groups according to the median intraoperative remifentanil dose received – low dose (≤ 0.215 µg/kg/min) and high dose (> 0.215 µg/kg/min) group.

Categorical data were presented as frequencies (percentages) and compared with the chi-square test. For continuous data, variables with normal distribution were expressed as mean ± standard deviation and compared with independent Student’s t-test, while variables with skewed distribution were compared with Mann-Whitney U test and expressed as median (Interquartile Range). Spearman’s correlation was used to determine the relationship between intraoperative remifentanil and postoperative morphine usage and pain score. A 2-sided p < 0.05 was considered statistically significant. Since all analyses were deemed to be exploratory, no adjustments were made for multiple tests of significance. All statistical analyses were performed using IBM SPSS Statistics for Macintosh, V22 (IBM Corp., Armonk, NY, USA).

## Results

A total of 142 patients were reviewed for eligibility from March 2019 until June 2020, and 97 patients were included in the analysis after excluding 45 patients in whom NRS record, or remifentanil dosage were incomplete or missing. The number of patients who received low (≤ 0.215 µg/kg/min) and high dose (> 0.215 µg/kg/min) of remifentanil were 41 and 56, respectively.

Patients’ demographic information was presented in Table 1. They were predominantly females (n = 78, 80.4%) with a mean age of 14.0 ± 2.0 years. The levels of vertebral fusion, Cobb angle, the number of screws used, and the length of skin incision were comparable between groups. The median remifentanil dose received in the low and high dose groups were 0.189 (0.169–0.202) µg/mg/min and 0.251 (0.228–0.276) µg/mg/min, respectively, which was significantly different (P < 0.001). The mean duration of remifentanil infusion was 134.9 ± 22.0 and 123.4 ± 23.7 min, respectively. The overall mean duration of remifentanil infusions was 127.3 ± 26.3 min.

**Table 1:** Demographic and perioperative characteristics

Variables	Total (N = 97)	Low dose (N = 41)	High dose (N = 56)	p-value
Age, years	14.0 ± 2.0	14.2 ± 2.0	13.9 ± 2.0	0.522
Sex				0.124
Male Female	19 (19.6) 78 (80.4)	11 (26.8) 30 (73.2)	8 (14.3) 48 (85.7)	
Height, cm	156.4 ± 8.6	158.9 ± 8.5	156.5 ± 8.2	0.011
Weight, kg	44.6 ± 9.3	49.9 ± 9.9	40.7 ± 6.6	< 0.001
BMI, kg/m^2^	18.2 ± 3.1	19.7 ± 3.7	17.0 ± 1.80	< 0.001
Cobb Angle, °, median (IQR)	58.5 (51.8–71.0)	59.0 (52.5–78.5)	63.0 (52.3–75.8)	0.841
Lenke Classification				0.125
1 2 3 4 5 6	41 (42.3) 18 (18.6) 3 (3.1) 1 (1.0) 24 (24.7) 10 (10.3)	22 (53.7) 7 (17.1) 2 (4.9) 1 (2.4) 7 (17.1) 2 (4.9)	19 (33.9) 11 (19.6) 1 (1.8) 0 17 (30.4) 8 (14.3)	
Number of vertebrae fused, median (IQR)	12.0 (10.8–13.0)	12.0 (11.0–13.0)	12.0 (11.0–13.0)	0.929
Number of screws, median (IQR)	14.0 (12.0–14.0)	14.0 (12.0–14.0)	14.0 (12.0–15.0)	0.883
Duration of surgery, minute	105.9 ± 21.5	110.9 ± 22.0	102.2 ± 20.5	0.049
Skin incision length, cm	29.4 ± 4.6	30.1 ± 4.5	29.0 ± 4.6	0.242
Estimated blood loss, mL	625.8 ± 315.2	658.6 ± 376.5	601.9 ± 262.5	0.384
Intraoperative remifentanil usage, µg	1247.7 ± 272.9	1232.3 ± 320.3	1259.0 ± 234.7	0.636
Intraoperative remifentanil usage, µg/kg	28.70 ± 7.24	24.79 ± 4.43	31.56 ± 7.60	< 0.001
Intraoperative remifentanil usage, µg/kg/min, median (IQR)	0.215 (0.191–0.241)	0.189 (0.169–0.202)	0.251 (0.228–0.276)	< 0.001
Duration of remifentanil infusions, minute	127.3 ± 26.3	134.9 ± 22.0	123.4 ± 23.7	0.017

*Note*: Data are presented as mean ± standard deviation or frequency (percentage), unless otherwise specified. The unit for remifentanil dose is µg/kg/min. Mean values for the following variables are Cobb angle: 65.3 ± 15.0°; vertebral level: 11.6 ± 2.0; number of screws: 13.5 ± 2.0; intraoperative remifentanil: 0.251 ± 0.259 µg/kg/min

Abbreviation: BMI, body mass index.

There were no significant differences in the pain score at rest and on movement between the two groups at every 6-hour interval up to 48 h post-operation (Tables 2 and 3 respectively). The mean pain score at rest and on movement at 48 h was comparable between these two groups (4.1 ± 1.0 [n = 40] vs. 4.1 ± 1.4 [n = 56]; P = 1.0; at rest, and 4.6 ± 0.9 [n = 41] vs. 4.8 ± 1.3 [n = 56]; P = 0.6; on movement).

**Table 2:** Numerical rating scale for pain score at rest at 6 hourly intervals

Postoperative time interval	PSAR (n)			P value
	Total (N = 97)	Low dose (N = 41)	High dose (N = 56)	
0–6 h	4.1 ± 1.7 (94)	4.4 ± 1.6 (39)	3.9 ± 1.7 (55)	0.173
6–12 h	4.3 ± 1.6 (92)	4.3 ± 1.3 (38)	4.3 ± 1.8 (54)	0.919
12–18 h	4.4 ± 1.7 (89)	4.4 ± 1.5 (35)	4.4 ± 1.8 (54)	0.951
18–24 h	4.2 ± 1.5 (92)	4.2 ± 1.2 (37)	4.2 ± 1.6 (55)	0.999
24–30 h	4.0 ± 1.4 (84)	4.1 ± 1.2 (32)	4.0 ± 1.6 (46)	0.953
30–36 h	3.9 ± 1.4 (78)	3.6 ± 0.9 (32)	4.1 ± 1.7 (46)	0.140
36–42 h	3.8 ± 1.6 (75)	3.8 ± 1.5 (31)	3.8 ± 1.7 (44)	0.900
42–48 h	3.7 ± 1.3 (55)	3.5 ± 1.2 (25)	3.9 ± 1.5 (30)	0.280
Mean over 24 h	4.3 ± 1.4 (96)	4.3 ± 1.1 (40)	4.2 ± 1.6 (56)	0.679
Mean over 48 h	4.1 ± 1.2 (96)	4.1 ± 1.0 (40)	4.1 ± 1.4 (56)	0.959

*Note*: Data are presented as mean ± standard deviation (number of patients). The unit for remifentanil dose is µg/kg/min

Abbreviation: PSAR, pain score at rest.

**Table 3:** Numerical rating scale for pain score on movement at 6 hourly intervals

Postoperative time interval	PSOM (n)			P value
	Total (N = 97)	Low dose (N = 41)	High dose (N = 56)	
0–6 h	5.0 ± 1.5 (97)	5.1 ± 1.4 (41)	4.9 ± 1.6 (56)	0.144
6–12 h	4.9 ± 1.4 (96)	4.9 ± 1.1 (41)	5.0 ± 1.6 (55)	0.693
12–18 h	5.0 ± 1.5 (97)	5.1 ± 1.5 (41)	5.0 ± 1.5 (56)	0.840
18–24 h	4.8 ± 1.5 (97)	4.8 ± 1.2 (41)	4.8 ± 1.6 (56)	0.849
24–30 h	4.7 ± 1.4 (94)	4.5 ± 1.3 (39)	4.8 ± 1.6 (55)	0.450
30–36 h	4.7 ± 1.3 (91)	4.4 ± 1.1 (36)	4.9 ± 1.5 (55)	0.091
36–42 h	4.4 ± 1.4 (84)	4.1 ± 1.4 (36)	4.5 ± 1.4 (48)	0.209
42–48 h	4.1 ± 1.2 (74)	4.0 ± 1.0 (34)	4.2 ± 1.4 (40)	0.514
Mean over 24 h	4.9 ± 1.3 (97)	5.0 ± 1.1 (41)	4.9 ± 1.4 (56)	0.868
Mean over 48 h	4.7 ± 1.1 (97)	4.6 ± 0.9 (41)	4.8 ± 1.3 (56)	0.583

Note: Data are presented as mean ± standard deviation (number of patients). The unit for remifentanil dose is µg/kg/min.

Abbreviations: PSOM, pain score on movement

The cumulative weight-adjusted morphine consumptions between these two groups also showed no significant difference when measured at 6-hour intervals up to 48 h (P = 0.122–0.919) (Table 4).

**Table 4:** Weight adjusted morphine consumption at 6 hourly intervals up to 48 h

Postoperative time interval	PCA morphine usage, mg/kg [n]			P value*
	Total (N = 97)	Low dose (N = 41)	High dose (N = 56)	
0–6 h	0.13 ± 0.13 0.09 (0.03–0.17) [97]	0.11 ± 0.10 0.10 (0.03–0.15) [41]	0.14 ± 0.15 0.08 (0.03–0.21) [56]	0.658
6–12 h	0.08 ± 0.07 0.06 (0.02–0.11) [97]	0.07 ± 0.07 0.05 (0.02–0.09) [41]	0.08 ± 0.07 0.06 (0.02–0.11) [56]	0.153
12–18 h	0.08 ± 0.08 0.06 (0.02–0.13) [97]	0.08 ± 0.08 0.04 (0.02–0.10) [41]	0.09 ± 0.08 0.06 (0.02–0.13) [56]	0.284
18–24 h	0.05 ± 0.07 0.04 (0-0.07) [97]	0.05 ± 0.05 0.04 (0-0.07) [41]	0.06 ± 0.07 0.04 (0-0.07) [56]	0.739
24–30 h	0.04 ± 0.05 0.02 (0-0.06) [94]	0.04 ± 0.04 0.02 (0-0.07) [41]	0.04 ± 0.06 0.02 (0-0.06) [53]	0.825
30–36 h	0.04 ± 0.05 0.02 (0-0.05) [86]	0.03 ± 0.04 0.01 (0-0.04) [37]	0.05 ± 0.06 0.02 (0-0.05) [49]	0.122
36–42 h	0.03 ± 0.05 0 (0-0.03) [45]	0.03 ± 0.06 0 (0-0.04) [23]	0.03 ± 0.05 0 (0-0.03) [22]	0.919
42–48 h	0.01 ± 0.03 0 (0-0.04) [8]	0.01 ± 0.02 0 (0-0.03) [5]	0.02 ± 0.04 0 (0-0.04) [3]	0.558
Total in 24 h	0.34 ± 0.27 0.27 (0.14–0.45) [97]	0.30 ± 0.23 0.26 (0.15–0.38) [41]	0.37 ± 0.30 0.27 (0.14–0.45) [56]	0.399
Total in 48 h	0.43 ± 0.31 0.37 (0.19–0.58) [97]	0.38 ± 0.25 0.38 (0.19–0.46) [41]	0.46 ± 0.35 0.37 (0.19–0.58) [56]	0.454

*Note*: Data are presented as mean ± standard deviation and median (interquartile range). The unit for remifentanil dose is µg/kg/min

^*^Mann-Whitney U test

Furthermore, when analysed using Spearman’s Rho correlation, no or negligible relationship can be established between intraoperative remifentanil usage with pain score and PCA morphine usage (spearman’s rho 0.04–0.129) (Table 5).

**Table 5:** Correlation between intraoperative remifentanil use with pain score and PCAM usage

	Spearman’s Rho	P value
Mean PSOM over 24 h	0.040	0.697
Mean PSOM over 48 h	0.096	0.348
Mean PSAR over 24 h	0.044	0.672
Mean PSAR over 48 h	0.105	0.307
Total PCAM usage in 24 h (mg/kg)	0.129	0.207
Total PCAM usage in 48 h (mg/kg)	0.113	0.269

*Abbreviation*: PSOM, pain score on movement; PSAR, pain score at rest; PCAM, patient-controlled analgesia morphine

## Discussion

In this retrospective analysis, we did not find any association between the different doses of remifentanil with the occurrence of postoperative hyperalgesia. There were no significant differences in the NRS for pain score at rest and on movement, and morphine consumption between the low and high-dose remifentanil groups.

The relationship between the use of remifentanil in clinical practice and the development of OIH remains controversial, despite the existence of many studies in the past. A review by Kim et al. [11] concluded that multiple issues, including but not limited to the dose and duration of opioids and methods used in assessing pain as the confounding factors. Adding to the gap is a lack of understanding and standard definitions for OIH, opioid tolerance, and withdrawal-associated hyperalgesia, which are often used interchangeably due to their overlapping symptoms [12]. Several methods have been suggested to evaluate the incidence of OIH in the clinical settings, including measuring pain intensity, opioid consumption, evaluation of secondary hyperalgesia using monofilaments and other sensory tests such as cold, heat, vibration, and electrical stimulation [5]. In our study, pain scores and morphine consumption were chosen given their practicality and frequent use in previous studies.

The result of our study is in concert with the finding of a recent observational study who found no association between their relatively high dose and long duration of intraoperative remifentanil (mean total remifentanil dose was 0.12 mg/kg, mean infusion duration was 435 min) and postoperative opioid consumption in adolescent idiopathic spine surgery when used in the context of propofol-based anaesthesia and multimodal analgesia [4]. In a similar type of surgery in adults, Yeom et al. [13] also found no evidence of acute opioid tolerance or hyperalgesia in patients undergoing spinal fusion despite a significant difference in their mean intraoperative infusion rate of remifentanil (0.16 vs. 0.03 µg/kg/min) and a more extended period of remifentanil infusion which is twice our mean duration (averaging 220 vs. 107 min). Likewise, in a placebo-controlled, double-blind study among healthy human volunteers, a long duration (3 h) of remifentanil TCI of up to 4.00 ng/ml also failed to establish the development of significant tolerance to analgesia [14]. The absence of tolerance with remifentanil infusion was also observed in twenty healthy male volunteers who received a 3-hour continuous infusion of remifentanil (0.08 µg/kg/min) in a randomized study [15].

The findings of our study, however, did not concur with the result of a randomized study in paediatric scoliosis surgery which elucidated that a mean remifentanil dose of 0.28 µg/kg/min was associated with a larger amount of cumulative morphine consumption, up to 30.0% greater than the intermittent morphine group at 24-hour after surgery [3]. This was supported by another randomized study [16] whereby intraoperative remifentanil of 0.40 µg/kg/min triggered significant hyperalgesia as well as a higher amount of morphine consumption for 48 h postoperatively when compared to the group that received lower remifentanil dose of 0.05 µg/kg/min. A more recent retrospective study identified that infusion of remifentanil of > 0.2 µg/kg/min increases the probability of treatment-requiring pain for 48 h after robotic thyroid surgery when adjusting for analgesic consumption and its interaction with time [17].

OIH has a complex underlying cellular mechanism that is poorly understood [18]. Nevertheless, N-Methyl-D-aspartate (NMDA) receptors have been frequently described as playing a central role in the development of OIH, which is supported by various experimental studies performed in both humans and animals [19]. In accord with this theory, the co-administration of sub-anaesthetic doses (0.5 mg/kg) of NMDA-receptor antagonist ketamine during induction in our study could explain the non-significant difference observed in the postoperative pain and morphine consumption among the groups. This observation was consistent with those of Joly et al. [3] in which the use of ketamine completely averted the undesirable increase in postoperative pain sensitivity and hyperalgesia that otherwise resulted from large-dose remifentanil infusion. Likewise, patients who received a large dose of remifentanil of 0.40 µg/kg/min with ketamine had remarkably less postoperative morphine requirement than those receiving the same remifentanil dose but without ketamine [3].

While ketamine has been frequently reported and used by clinicians to prevent OIH, little is known previously about the use of paracetamol in preventing this phenomenon. A prospective, randomized, placebo-controlled trial comparing the effect of ketamine and paracetamol in ninety patients undergoing total abdominal hysterectomy concluded that the latter is as effective as the former in preventing remifentanil-induced hyperalgesia [20] In accord with this study, the use of pre-emptive intravenous paracetamol could have diminished the effects of hyperalgesia, and its co-administration with ketamine as described above could make the undesirable OIH even less pronounced in our study.

Furthermore, the implementation of multimodal analgesia in our routine practice could have improved pain control and reduced the need for morphine postoperatively. The use of multimodal analgesia has been proven to be equivalent to the conventional PCA for acute postoperative pain management in patients who underwent one or two-level posterior lumbar fusion surgery [21]. All our patients received regular oral paracetamol and cyclooxygenase-2 (COX-2) inhibitor (Celecoxib) in the postoperative period until discharge. As the use of opioids in the postoperative period can lead to the vicious cycle of OIH by activating the astroglia and microglia in the central nervous system [22], the use of opioid-sparing strategies are believed to be the most effective way of preventing OIH [23].

The use of COX-2 inhibitor is supported by a crossover study, in which pre-treatment with both parecoxib and ketorolac was found to reduce the area of hyperalgesia following remifentanil infusion, whereby more excellent effects were observed with selective COX-2 inhibition (parecoxib) than COX-1 inhibition (ketorolac) [24]. As both the enzymes are present in the spinal cord, their inhibition prevents glutamate stimulation that would otherwise lead to NMDA activation. Thus, it is plausible that systemic administration of COX inhibitors reduces OIH by direct action at the spinal cord level [25].

The divergent findings of OIH incidence in studies using remifentanil could be partly explained by the differences in the cumulative remifentanil dose used intraoperatively. A smaller cumulative dose of remifentanil with a mean of 28.70 ± 7.24 µg/kg in our study was considered insufficient to elicit hyperalgesia reliably, as suggested by Angst [2]. According to his analysis, the increase in opioid consumption and pain score was consistently reported in those studies in which the cumulative remifentanil dose was greater than 50 µg/kg and could not be reliably detected if smaller doses were administered.

In addition, the relatively shorter duration of remifentanil use in our study (mean 127.3 ± 26.3 min) could have attenuated the OIH and explained the reduced intensity in pain score and the lesser cumulative morphine consumption in the high dose remifentanil group. In a comparative study [13] using remifentanil as an adjuvant in general anaesthesia with sevoflurane or propofol in adults undergoing spinal fusion, Yeom et al. have failed to exhibit evidence of hyperalgesia and has concluded that their short duration of remifentanil infusion (averaging 216 min in sevoflurane/remifentanil group and 225 min in propofol/remifentanil group) as confounding factors. A recent and similar retrospective study [4] in adolescent idiopathic spine surgery also found no association between their long duration of remifentanil infusion with postoperative opioid consumption, despite the mean duration of 435 min, which is four times longer than ours.

Our study has several strengths. Our sample size is relatively large as compared to other similar retrospective studies done previously [4, 26]. Our patient’s selection was only in AIS patients, with the mean age of 14.0 ± 2.0 years, and recruited from a single tertiary institution to ensure homogeneity of our data. Our study also has several limitations. The retrospective nature of our research was subjected to registry bias when retrieving the data, in addition to the absence of a proper comparison or control group. For instance, the distribution of the Lenke curve types were not equal where there were more Lenke 5 and 6 curves in the high dose remifentanil group. Nonetheless, the surgical strategy in terms of instrumentation and correction were still similar in all Lenke curve types and in our opinion might not be an important confounding factor for the outcome of this study. The retrospective design also prevents accurate evaluation and diagnosis of hyperalgesia. The result of this study should not be over-interpreted and should be considered as hypothesis-generating due to the exploratory nature and the lack of power analysis. Ideally, a prospective randomized trial using clear separation of remifentanil dose (low dose versus high doses) should be designed in the future to study the causal relationship between remifentanil and opioid-induced hyperalgesia. The use of a sub-anaesthetic dose of ketamine, paracetamol, and postoperative multimodal analgesia could have been the confounding variables affecting the outcome. However, these were necessary and considered the current standard of care.

## Conclusions

In conclusion, based on this retrospective analysis, no association was found between the use of intraoperative remifentanil and postoperative hyperalgesia in AIS surgery. These results support our current practice of using short duration of clinically useful doses of remifentanil infusion combined with multimodal analgesia. However, more robust study is required to confirm our findings.

## Data Availability

The data to support the results of this study is available from the corresponding author on reasonable request.

## Declarations

The authors have presented the abstract at the Annual Scientific Congress 2021 organised by the Malaysian Society of Anaesthesiologists & College of Anaesthesiologists and it has been published as a supplement of the UKM’s Medicine and Health Journal [27].

## Abbreviations

AIS: Adolescent idiopathic scoliosis
ASA: American Society of Anesthesiologists
BMI: Body mass index
IV: Intravenous
NMDA: N-Methyl-D-aspartate
NRS: Numerical rating scale
OIH: Opioid-induced hyperalgesia
PACU: Post-anaesthesia care unit
PCA: Patient-controlled analgesia
PSAR: Pain score at rest
PSOM: Pain score on movement
TCI: Target-controlled infusion
TIVA: Total intravenous anaesthesia
